# Participation and chronic illness: Nurses’ perceptions in primary care in Brazil, Germany, and Spain: Marcus Heumann

**DOI:** 10.1093/eurpub/ckac129.721

**Published:** 2022-10-25

**Authors:** M Heumann, G Röhnsch, E Zabaleta-del-Olmo, B Rosana Gonçalves de Oliveira Toso, L Giovanella, K Hämel

**Affiliations:** 1 Health Services Research and Nursing Science, School of Public Health, Bielefeld University, Bielefeld, Germany; 2 Qualitative Social and Education Research, Freie Universität Berlin, Berlin, Germany; 3 IDIAP JORDI GOL, Barcelona, Spain; 4 Centre for Biology and Health Research, Western Paraná State University, Cascavel, Brazil; 5 National School of Public Health, Oswaldo Cruz Foundation, Rio de Janeiro, Brazil

## Abstract

**Background:**

Many chronically ill persons are challenged by integrating the illness in everyday life and making ‘competent’ decisions on their life and care. In multiprofessional primary care, promoting clients’ self-management and strengthening their abilities to participate in everyday life is increasingly recognized as a nursing task. This study investigates facilitating and inhibiting conditions that nurses experience when exercising this task.

**Methods:**

Drawing upon a phenomenological approach, we conducted guided interviews with 34 practicing nurses and 23 key informants with advanced knowledge of primary health care nursing practice in Brazil, Germany, and Spain. The interviews were analysed using structuring content analysis.

**Results:**

The interviewees see competencies of nurses to establish trusting relationships with chronically ill clients as key to greater client participation. Nurses, however, state that bonding with clients can be time-consuming and exhausting. They consider it fundamental that physicians and other professionals value nurses’ efforts towards stronger client participation as a way forward to reach for person-oriented primary care. They criticize that especially physicians value biomedical tasks more than enabling participation. Referring to primary health care organisation, nurses experience that pressure of time through a growing number of routine and administrative tasks inhibits their efforts to strengthen clients’ participation.

**Conclusions:**

To promote the participation of clients with chronic illnesses in their everyday life and in care, relationship building with clients and self-management support needs to be acknowledged as an important scope of practice approached by nurses. To be able to unfold the potentials nurses need to be equipped with sufficient time and skills.

